# Evaluation and Predictive Modeling of Removal Condition for Bioadsorption of Indigo Blue Dye by *Spirulina platensis*

**DOI:** 10.3390/microorganisms8010082

**Published:** 2020-01-07

**Authors:** Felipe Robledo-Padilla, Osvaldo Aquines, Arisbe Silva-Núñez, Gibrán S. Alemán-Nava, Carlos Castillo-Zacarías, Ricardo A. Ramirez-Mendoza, Ricardo Zavala-Yoe, Hafiz M. N. Iqbal, Roberto Parra-Saldívar

**Affiliations:** 1 Department of Physics and Mathematics, Universidad de Monterrey, Av. Morones Prieto 4500, San Pedro Garza García 66238, N.L., Mexico; felipe.robledo@udem.edu (F.R.-P.); osvaldo.aquines@udem.edu (O.A.); 2 Tecnologico de Monterrey, School of Engineering and Sciences, Campus Monterrey, Ave. Eugenio Garza Sada 2501, Monterrey CP 64849, N.L., Mexico; arisbesilva@gmail.com (A.S.-N.); gibran.aleman@gmail.com (G.S.A.-N.); carloscastilloz@tec.mx (C.C.-Z.); ricardo.ramirez@tec.mx (R.A.R.-M.); 3 Tecnologico de Monterrey, School of Engineering and Sciences, Campus Mexico City, Calzada del Puente 222, Col. Ejidos de Huipulco, Mexico City 14380, Mexico; rzavalay@tec.mx

**Keywords:** indigo blue dye, *Spirulina platensis*, dye removal, linear modeling

## Abstract

Among the different chemical and physical treatments used to remove the color of the textile effluents, bioremediation offers many benefits to the environment. In this study, we determined the potential of *Spirulina platensis* (*S. platensis*) for decolorizing indigo blue dye under different incubation conditions. The microalgae were incubated at different pH (from 4 to 10) to calibrate for the optimal discoloration condition; a pH of 4 was found to be optimal. The biomass concentration in all experiments was 1 g/L, which was able to decolorize the indigo blue dye by day 3. These results showed that *S. platensis* is capable of removing indigo blue dye at low biomass. However, this was dependent on the treatment conditions, where temperature played the most crucial role. Two theoretical adsorption models, namely (1) a first-order model equation and (2) a second-order rate equation, were compared with observed adsorption vs. time curves for different initial concentrations (from 25 to 100 mg/L). The comparison between models showed similar accuracy and agreement with the experimental values. The observed adsorption isotherms for three temperatures (30, 40, and 50 °C) were plotted, showing fairly linear behavior in the measured range. The adsorption equilibrium isotherms were estimated, providing an initial description of the dye removal capacity of *S. platensis*.

## 1. Introduction

In recent years, the presence of micro and nanopollutans in water resources has become a common challenging issue for water and wastewater treatment processes. These pollutants consist of natural and human waste substances, which include pharmaceuticals, personal care products, steroid hormones, industrial chemicals, heavy metals, radioactive elements, pesticides, and many other emerging compounds. The term is derived from the substance concentrations ranging from a few ng/L to several μg/L [1,2].

The micropollutants are not affected by the usual biological treatment. In the wastewater treatment plants, the dissolved pollutant transfer from source to the environment in accumulative process and eventually the consumption of this water represents a minor and major health risks, toxicity, and more [3].

Nevertheless, recent progress has been made in an integrated perspective of wastewater treatment that includes standard and biological technologies for pharmaceutical emerging contaminants [4,5,6,7], industrial chemicals [8,9], brewery wastewater [10], agro-industrial wastewater [11,12], heavy metals [13,14], and emerging compounds [15].

The textile industry is the largest consumer of dyes, which are hard to degrade due to their high stability in various environmental conditions [16]. This industry represents about 31% of the world market, and one of the most essential dyes used in the industry is indigo blue, which accounts for 7% of the dyes used [17]. Industrial growth has increased the volume of waste discharged to water bodies, including substances that severely biodegrade, such as the dyes used in the textile industry, many of which are toxic or carcinogenic [18,19].

To remove the color of the textile effluents, sometimes different chemical treatments are used, including physical and biological agents [20,21,22,23,24]. However, some of these methods are impractical for application due to their high costs or waste production. Some techniques that are efficient but expensive have been reported, such as the use of activated charcoal [25], which has been implemented in small industries [26]. Nevertheless, these techniques also present several disadvantages, such as high reagent, high energy consumption, and low kinetics [27].

The treatment of textile wastewater containing indigo dye is one of the most difficult because the dye is of synthetic origin and has a molecular structure aromatic complex, which is more stable and harder to biodegrade [27,28,29]. The alteration of their chemical structures can result in the formation of new compounds xenobiotics, which may be more toxic than the potential compounds [30].

A variety of removal techniques have been used for this dye [25], including bioremediation using species of fungi or microalgae [31,32,33] and bacterial consortium [34]. The use of microorganisms benefits the environment since they have the potential to transform the contaminants into other less harmful substances or reduce their harmfulness [35,36,37]. Microalgae can remove a diverse number of dyes; among the species that have been the most studied are *Chlorella vulgaris*, *Cosmarium sp., Scenedesmus quadricauda*, *Scenedesmus* sp., *Spirogyra species*, *Phitosphora* sp., and *Desmodesmus sp.* [24,26,38,39,40,41]. This removal is carried out by the biosorption process by the several functional groups that can form complexes with dyes through physicochemical interactions [42].

However, the removal of the blue dye indigo has only been tested with *Scenedesmus quadricauda* [34]. In this work, we examined the potential of the microalga *S. platensis*, which is rich in carbohydrates and proteins, to remove the dye. We found that *S. platensis* is capable of removing indigo blue dye at low biomass under different temperature and pH conditions.

Nowadays, biotechnological processes need to be approached from a multidisciplinary perspective, where physics and mathematics are fundamental tools for understanding the underlying processes of chemical and biological phenomena. The use of more powerful computational tools is standard, and statistical programs facilitate the incorporation of more robust predictions for understanding these processes. An adsorption isotherm is a prediction that describes the capture and retention of diluted dye in the water at a constant temperature and pH. An isotherm is an essential tool used to describe and predict the mobility of this substance in aqueous environments [43].

The isotherms depict graphically the mathematical correlation between concentrations, temperature, pH, and other physicochemical parameters, which plays an important role in the modeling analysis, operational design, and application of the adsorption systems, expressing the solid-phase against its residual concentration [44]. These parameters and the thermodynamic assumptions of the particular problem are used to get insight into the adsorption mechanism, surface properties, and the degree of affinity of the adsorbents [45].

On the other hand, the kinetic models describe the reaction order of adsorption systems as a function of the solution concentration and the capacity of the adsorbent. Some of these kinetic models are the first-order and second-order reversible, first-order and second-order irreversible, and pseudo-first-order and pseudo-second-order models, Lagergren’s first-order equation, Zeldowitsch’s model, and Ho’s second-order expression [46].

In this study, we examined the mathematical correlation between experimental data and isotherm curves, starting from a simple geometrical model of diffusion to obtain the first-order classical kinetic model. A second-order model is presented for comparison of the results. Both models showed similar accuracy and presented a good approximation to experimental values. Similarly, adsorption equilibrium isotherms were estimated, providing an initial description of the dye removal capacity of the algae.

## 2. Materials and Methods 

### 2.1. Inoculum Preparation

A calibration curve was created with a stock solution of indigo blue synthetic dye (Sigma-Aldrich, San Luis, MO, USA) for concentrations ranging from 50 to 800 mg/L. Then, the optical density was measured at 600 nm by UV-Vis spectrophotometer (HACH, Loveland, CO, USA) [47]. For the determination of the point of zero charge (pHzpc), 25 mL solutions of microalgae were prepared at different pH (3, 5, 7, and 9) in triplicate, each flask was incubated at 140 rpm and 25 °C, and after 24 h underwent pH measurement. We analyzed pH (4, 5, 6, 7, 8, 9, and 10), dye concentration (6.25,12.5, 25, 50, and 100 mg/L), temperature (30, 40 and 50 °C), and time (0 to 4 days). Each one of the tests was conducted by dissolving the lyophilized microalga in distilled water to the concentration of 1 g/L.

All tests were performed in triplicates by incubating for 24 h at 3000 rpm in an incubator (Shel Lab, Orlando, FL, USA). All the samples were centrifuged at 10,000 rpm in a centrifuge prism (Thermo scientific, Waltham, MA, USA) for 2 min at the time of measuring the optical density. 

### 2.2. Experimental Design

To define the optimum pH, we prepared 12.5 mL solutions of microalgae at different pH (4, 5, 6, 7, 8, 9, and 10) in triplicate in tubes. Simultaneously, 12.5 mL solutions of indigo blue at a concentration of 50 mg/L were processed at the same pH. A 1 mL sample was taken from each tube was centrifuged at 10,000 rpm for 2 min, and we measured the optical density of the supernatant. After, all the tubes were incubated at 3000 rpm for 24 h. The resultant samples were used to measure the optical density and pH to determine the highest removal rate.

To measure the effect of time and concentration, around 12.5 mL solution of microalgae was prepared at optimum pH. In tubes, we processed 12.5 mL solutions of indigo blue at concentrations of 25, 50, 75, and 100 mg/L were processed at the same pH. A 1 mL sample was removed from each tube, then centrifuged at 10,000 rpm for 2 min, and we measured the initial optical density of the supernatant. Subsequently, all the tubes were incubated at 3000 rpm for 4 days and we removed samples at the same time each day to measure the respective optical density. 

To measure the effect of temperature, we also used 12.5 mL solutions of microalgae at optimum pH. Similarly, we prepared 12.5 mL solutions of indigo blue in tubes at concentrations of 6.25, 12.5, 25, 50, and 100 mg/L at the same pH. From each of the tubes, we removed a 1 mL sample, which was then centrifuged at 10,000 rpm for 2 min, and we then measured the optical density of the supernatant. Afterward, all the tubes were incubated at 3000 rpm for 4 days. The experiment was conducted consistently at 30, 40, and 50 °C.

The removal percentage is calculated according to the following formula:

(1) $$D=\frac{100\;\times \;\left(C_{i}-C_{t}\right)}{C_{i}},$$

where *D* is the discoloration of the indigo blue (%), *C_i_* is the initial concentration (mg/L), and *C_t_* is the concentration over time (mg/L).

The total adsorption is calculated as follows:

(2) $$Q=C_{i}-C_{t},$$

where *Q* is the total absorbed quantity of indigo blue (mg/L).

Finally, the adsorption capacity is:

(3) $$q=\frac{Q}{B},$$

where *q* (mg/g) is the adsorption capacity, and *B* is the concentration of biomass (g/L), which was consistently 1 g/L for all experiments. The study conditions are summarized in Table 1.

### 2.3. Mathematical Models

We first used a geometrical model for the diffusion equation, and we assumed that *S. platensis* could be modeled as a large cylinder with a radius much less than its length [48]. The diffusion equation can be expressed as:

(4) $$\frac{1}{r}\;\times \;\frac{\partial C}{\partial r}\left(r\;\times \;\frac{\partial C}{\partial r}\right)=\frac{1}{\alpha }\;\times \;\frac{\partial C}{\partial t},$$

where *C* is the equilibrium concentration (mg/L), *α* is the rate constant (h^−1^), *r* is the radius, and *t* is the time (h).

Equation (4) can be solved by separation of variables with solution C(r,t)=R(r) × C(t), where the radial solution R(r) can be obtained from the differential equation:

(5) $$r^{2}\;\times \;\frac{d^{2}R}{dr^{2}}+r\;\times \;\frac{dR}{dr}+\gamma^{2}\;\times \;r^{2}\;\times \;R=0$$

where *γ* is the constant of separation, and the temporal solution comes from C(t) = C_t_, that is,

(6) $$\frac{dC_{t}}{dt}+\alpha \;\times \gamma^{2}\;\times C_{t}=0$$

Equation (5) is solved using the Bessel equation of the first kind of order zero. However, in this work, the adsorption overtime was more important rather than the radial adsorption in *Spirulina*. For this reason, our analysis focused on Equation (6), which represents the first-order classical kinetic model of total adsorption [43]. Its solution is:

(7) $$C_{t\left(1\right)}=C_{i}\;\times \;\exp\left(-\alpha \gamma^{2}t\right),$$

where $C_{i}$ is the initial concentration of the solution of the blue indigo dye. The rate constant *α* is related to the adsorption capacity or how fast the dye solution is transferred to the *Spirulina* cells. The constant γ is related to the radial adsorption capacity of the *Spirulina* cylindrical geometry. 

A second-order model presented by Ho [46] was adopted as an alternative approach to comparing the first-order model. This is a linear form of the typical second-order rate equation:

(8) $$\frac{1}{C_{t\left(2\right)}}=k_{2}\times t+\frac{1}{C_{i}},$$

where *C_t(2)_* is the concentration (mg/L), *C_i_* is the initial concentration (mg/L), *t* is time (h), and *k_2_* is the rate constant (L/(mg h)) or how fast the dye solution is transferred to the *Spirulina* cells.

### 2.4. Statistical Analysis

Experimental results were analyzed using R 3.3.3 (R Foundation for Statistical Computing, Vienna, Austria) using a significance of 95%. Linear regression was used for isotherms, whereas adsorption vs. time curves were fitted with nonlinear regression. Therefore, in these cases, the root mean squared error (RMSE) or residual standard error (RSE) is provided instead of the coefficient of determination (*R^2^*) to compare the quality of the fit. In all cases, the fitted parameters were found to be statistically significant. The model selection criterion (MSC) [49] was applied to compare the fitted models.

## 3. Results and Discussion

Concentrations of the dye were measured via optical density (OD). The standard curve of indigo blue can be expressed using a linear function, OD = *m × C + b*, which is OD = 0.003*C* + 0.0348, where OD is the optical density at 60 nm and *C* is the concentration (mg/L). The *R^2^* of the curve was of 0.9989. 

### 3.1. Optimum pH

**Figure 1:**
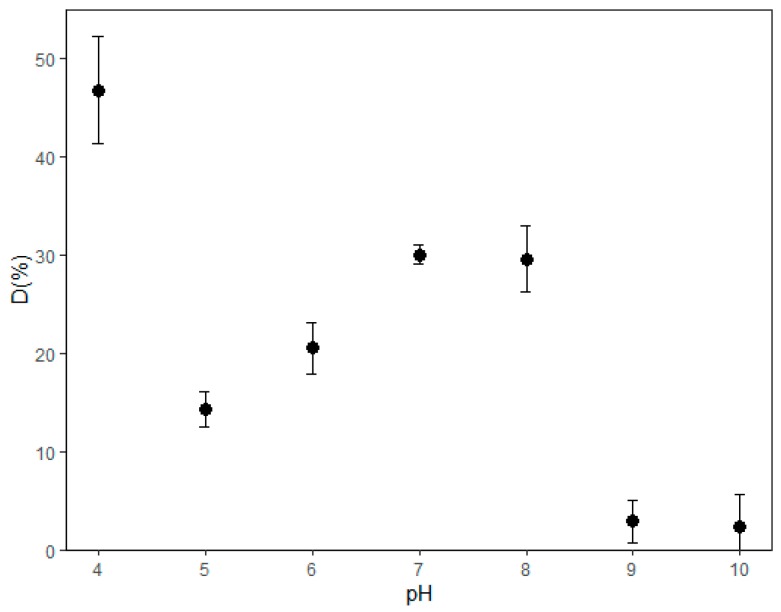
Indigo blue dye removal percentage (D from Equation (1)) at different levels of pH.

Adsorption measurements were recorded at different pH values after 24 h to establish the optimum operating conditions. Figure 1 shows the measured removal percentages as a function of pH. The maximum removal of 46.84% was achieved at pH 4, which was determined to be the optimum pH for this process. It is possible that the protonation of some functional groups of the dye is present. At pH 5, you can have less load, so it will be more complicated to have high adsorption. This phenomenon may not occur in the other tested pH. Therefore, the remainder of the experiments were conducted at a pH of 4.

This result contrasts those reported in previous articles [34], where the authors analyzed the optimal pH of the microalga *Scenedesmus* and identified pH 7.5 as optimal for discoloring the indigo blue. Petrović et al. [50] reported an optimal pH of 2.0 for a new biosorbent, synthesized by chemically modifying *Lagenaria vulgaris* shell with ZrO_2_ and abbreviated as LVB-ZrO_2_ with blue textile dye RB19. 

Once pH was measured after 24 h, the pHzpc was obtained at 7.7 of pH for *Spirulina*. In contrast again, for LVB-ZrO_2_ with RB19 dye, a pHzpc of 5.49 was determined.

### 3.2. Measurement of the Effect of Time and Concentration

**Figure 2:**
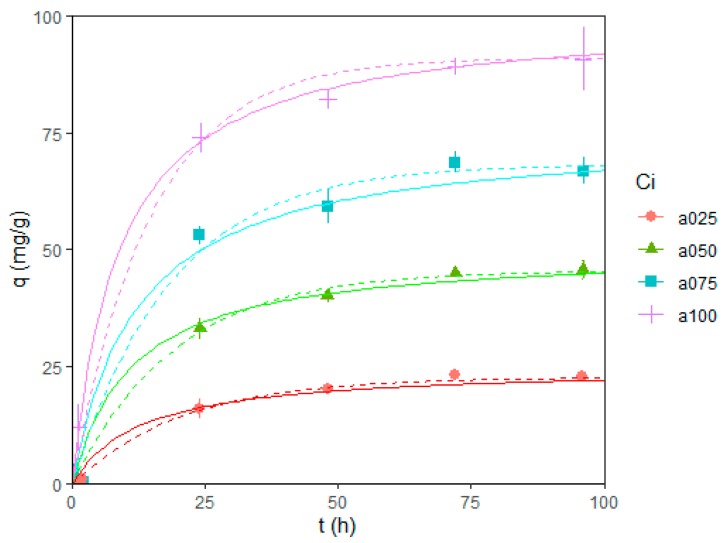
Adsorption curves of blue indigo dye for different initial concentrations *C_i_* (25, 50, 75, and 100 mg/L). Points with error bars represent measurements with experimental errors. Dashed lines represent a fit to the first-order model in Equation (7). Solid lines represent a fit to the second-order rate in Equation (8) [46], q is the adsorption capacity of the microalgae, and t is the time in hours.

The amount of absorbed dye as a function of time was measured for four different initial concentrations of indigo blue (25, 50, 75, and 100 mg/L) for 96 h. Figure 2 shows the observed experimental values (solid dots with error bars) and two fitted models for each initial concentration, where the dashed line is the first-order model in Equation (7) and the solid line is the second-order rate model in Equation (8) [46]. Only one parameter was fitted to have more meaningful parameter values in all models [41,49,50]. 

For the first-order model, the fitted parameter was the adsorption rate, and the maximum adsorption (*Q_e_*) was fixed to 91% of the initial concentration (*C_i_*) since we observed that the adsorption process reached equilibrium after three days and the amount of dye absorbed was consistently around an average of 91% of the initial concentration (*C_0_*) in all cases (Table 2).

In the second-order model, the only parameter is the rate constant. Table 3 shows the fitted values for each concentration and model combination. Since the models are not linear, *R^2^* values are not obtained; instead, the RMSE or RSE of the fit is presented.

To compare between models, the MSC [49] was applied by assigning equal weights to all measured data points:

(9) $$MSC=\ln\left(\frac{\sum_{j=1}^{d}w_{j}{\left(C_{j}-\bar{C}\right)}^{2}}{\sum_{j=1}^{d}w_{j}{\left(C_{j}-C_{m_{j}}\right)}^{2}}\right)-\frac{2\lambda }{d},$$

where *d* is the number of data points, *λ* is the number of fitted parameters, *C_j_* is the *j*th observed concentration from the replicate experiments, $\bar{C}$ is the average of observed concentrations, $C_{m_{j}}$ is the model calculated concentration corresponding to the *j*th replication, and *w_j_* is a weighting factor (optional).

For the first-order model, rate constants increase with larger initial concentrations of dye. In contrast, for the second-order model, rate constants decrease with larger initial concentrations of dye (within statistical error), behaving more consistently with similar studies of contaminant adsorptions of algae [51]. Despite this inconsistency, both models have good predictability (>90%, as seen from the observed residual standard errors). The estimated MSC values show that both models have similar accuracies at all initial concentrations, but the first-order model is consistently slightly better.

### 3.3. Measurement of the Effect of the Temperature and Concentration

**Figure 3:**
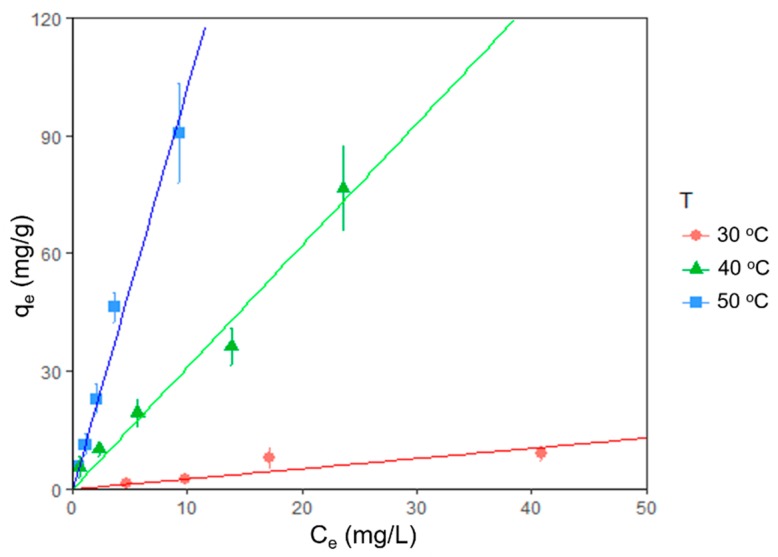
Isotherm plots showing temperature effect on dye removal. Final adsorption (q_e_) is plotted vs. final concentration (C_e_) at three different temperatures (30, 40, and 50 °C). Points with error bars represent measurements with the experimental error. Lines represent fit with the theoretical model linear isotherms.

The effect of temperature on the adsorption was analyzed plotting equilibrium adsorption isotherms for 30, 40, and 50 °C, where *q_e_* (Equation (1)) on the vertical axis is the amount of dye absorbed in the biomass at equilibrium and *C_e_* on the horizontal axis represents the amount of dye remaining in the solution at equilibrium (all measurement were recorded after four days). Figure 3 shows that the removal of the blue indigo dye is more effective as temperature increases within the experimental range. For this case, the maximum adsorption was observed at 50 °C, where the amount of absorbed dye was consistently 10.22 times the amount remaining in solution at equilibrium. We found no sign of saturation in the range of concentration and temperature examined. All 30, 40, and 50 °C isotherms were fairly linear type C within the measured range [43].

Table 4 shows the obtained values for a linear fit with an intercept of the origin (*q_e_ = b_o_C_e_*) for each isotherm of Figure 3, where *b_o_* represents the slope of the linear model, where the initial concentrations were 6.25, 12.5, 25, 50 and 100 mg/L.

The strong correlation (fit) for each isotherm shows the linearity of the measured range, where the high dependence on the temperature suggests a chemical adsorption process. Furthermore, from 40 to 50 °C, we see a significant change in the order of magnitude of the slope *b_o_*, which indicates the process needs more energy for a more efficient reaction. In contrast, for *Chlorella vulgaris* [51], the maximum adsorption equilibrium is reached at lower temperatures (25 and 35 °C), suggesting a different mechanism of adsorption for both species.

## 4. Conclusions

Our findings prove that despite having lyophilized biomass, physicochemical conditions are essential when trying to remove indigo blue dye from the solution.

The optimum operating pH was found to be 4 with high variations. This means that the conditions under which the algae perform the removal may vary depending on the species or the conditions under which the dye is tested. 

In the experiment, the measured range equilibrium absorptions were consistent around 91%, showing no saturation; however, similar measurements in the literature show saturation at going to significantly higher initial concentrations.

In terms of the temperature, the isotherms were fairly linear in the measured range, and we observed that the removal capacity increases with temperature within the experimental range (30–50 °C) regardless of the initial dye concentration. Similar measurements reported in the literature are as follows: Azu and Tezer [51] observed nonlinear behavior in the absorption isotherms of Remazol Black B (RB) dye by *Chlorella vulgaris*. However, the measurements were recorded in a significantly wider dye concentration range. Physical adsorption models provide good predictability (<90%) in adsorption vs. time curves. Both first- and second-order models show similar accuracies, but the first-order model is consistently better (as shown by estimated MSC and residual values) independent of the initial concentrations. In contrast, similar measurements reported in the literature [51] better fit the second-order model.

Other dye absorption measurements, such as methylene blue onto activated carbon [52], reach saturation at *C_e_* = 50 mg/L and isotherms have to be modeled non-linearly. The adsorbent concentration in that experiment was five times smaller (0.2 g/L) than in ours. 

When compared to the absorption of dye onto fly ash, this measurement was more stable, maintaining an average absorption of 91% for dye concentrations of 25 mg/L to 100 mg/L, whereas the measurement with ash shows an early saturation [53]. The removal of dye onto ash decreases from 98.7% to 69.1% when the initial dye concentration increases from 5 to 20 mg/L. Similarly, in this measurement, the first-order model more accurately describes the absorption vs. time.

The adsorption process reaches equilibrium after four days. Since there is no observed saturation, in the measured range the dominant process is chemical adsorption. Despite the linearity at the measured range, the high dependence on the temperature suggests a physicochemical interactions, as mentioned previously [42].

Only two species of microalgae have been tested for the removal of the indigo blue (*Spirulina platensis* here and *Scenedesmus quadricauda* [15]). Both were able to achieve removals between 90% and 100%. However, in both cases, the tests ran for three to four days, so experiments with other species are recommended to see if any can remove more dye in fewer days. Similarly, for methylene blue, *Desmodesmus sp.* has a 98.6% removal after six days [24]. 

For other dyes and microalgae species, lower removal efficiency has been reported. For example, for AB-161 dye from tannery effluent, *Scenedesmus sp.* achieved a removal of 69.83% [41]. For malachite green, *Cosmarium sp.* has removal of 74% [26].

Further work can be done in the future of scaling up these results. The process was planned solely for laboratory testing, as a proof of concept. It would be necessary to scale the algae to at least 1000 L, which is something that would imply explaining how the bioreactor in which it would grow would work. For example, the agitation of 3000 rpm was to incubate the algae with the dye; at large levels, this agitation would have to be replaced by aeration, which would make the process extremely expensive. Similarly, to achieve the maximum absorption at 50 °C of the dye would imply a high cost.

We also recommend investigating the influence of the number of lipids, proteins, and carbohydrates present in the microalgae and how these nutrients increase or decrease the load on the cell wall of the biomass.

## Figures and Tables

**Table 1:** Experimental conditions for experimental designs. The pH was fixed at 4 after optimum pH test. The concentration of *S. platensis* was of 1 g/L for all test.

Experimental Design	pH	Indigo Blue Dye Concentration (mg/L)	Time (Hours)	Temperature (°C)
Optimum pH	4, 5, 6, 7, 8, 9, and 10	50	24	25
Time and concentration	4	25, 50, 75, and 100	0, 24, 48, 72, 96	25
Temperature and concentration	4	6.25, 12.5, 25, 50, and 100	96	30, 40, and 50

**Table 2:** Observed and expected total adsorptions for different initial concentrations. The expected value of the adsorption was 91% of the initial concentration taken from the average equilibrium adsorption measurements after three days.

*C_i_* (mg/L)	Expected *Q_e_* (mg/L) = 0.91*C_o_*	Observed Average *Q_e_* (mg/L) (95% *C_i_*)
25	22.8	23.1 ± 0.3
50	45.5	45.4 ± 2.3
75	68.3	67.7 ± 4.5
100	91	89.9 ± 7.21

**Table 3:** Fitted parameters for adsorption vs. time curves at different initial concentrations (25, 50, 75 and 100 mg/L): first-order model in Equation (7) and a second-order rate equation (Equation (8)) [46]. (SE for square error, RMSE for root-mean square error, MSC for model selection criterion).

*Ci* (mg/L)	First-Order Rate Constant *K1* (1/h) (Estimate ± SE)	First-Order Model Residual SE RMSE	First-Order Residual MSC	Second-Order Rate Constant *K2* (L/(mg·h)) (Estimate ± SE)	Second-Order Model Fit Residual SE RMSE	Second-Order Fit MSC
25	0.046 ± 0.0047	1.146	5.10	0.00265 ± 0.00050	1.954	4.15
50	0.052 ± 0.0027	0.993	6.03	0.00180 ± 0.00013	1.128	5.79
75	0.047 ± 0.0081	5.64	4.60	0.00088 ± 0.00022	7.648	4.02
100	0.067 ± 0.015	8.774	4.14	0.00095 ± 0.00021	8.722	4.15

**Table 4:** Values for a linear fit with an intercept of the origin for each isotherm.

Temperature	*b_o_* (L/g) (Estimate ± SE)	*p*-Value	*R^2^* (fit)
30 °C	0.260 ± 0.047	0.011	0.883
40 °C	3.104 ± 0.1622	<0.001	0.987
50 °C	10.220 ± 0.500	<0.001	0.988

## Abbreviation

*α*	rate constant (h^−1^)
*B*	concentration of biomass (g/L)
*C*	equilibrium concentration (mg/L)
*C_e_*	amount of dye left in the solution at equilibrium (mg/L)
*C_i_*	initial concentration (mg/L)
*C_t_*	concentration over time (mg/L)
$\bar{C}$	observed average concentrations
*D*	discoloration of the indigo blue (%)
*k_2_*	rate constant (L/(mg·h))
*Q*	total adsorption (mg/L)
*q*	adsorption capacity (mg/g)
*q_e_*	amount of dye absorbed in the biomass at equilibrium (mg/g)
*t*	time (h)
